# Inorganic polyphosphate is produced and hydrolyzed in F_0_F_1_-ATP synthase of mammalian mitochondria

**DOI:** 10.1042/BCJ20200042

**Published:** 2020-04-29

**Authors:** Artyom Y. Baev, Plamena R. Angelova, Andrey Y. Abramov

**Affiliations:** 1 Laboratory of Experimental Biophysics, Centre for Advanced Technologies, Tashkent, Uzbekistan; 2 Department of Clinical and Movement Neurosciences, UCL Queen Square Institute of Neurology, London WC1N 3BG, U.K.; 3 Sechenov First Moscow State Medical University, 119048 Moscow, Russia

**Keywords:** bioenergetics, F_0_-F_1_-ATPase, inorganic polyphosphates, mitochondria

## Abstract

Inorganic polyphosphate (polyP) is a polymer present in all living organisms. Although polyP is found to be involved in a variety of functions in cells of higher organisms, the enzyme responsible for polyP production and consumption has not yet been identified. Here, we studied the effect of polyP on mitochondrial respiration, oxidative phosphorylation and activity of F_0_F_1_-ATPsynthase. We have found that polyP activates mitochondrial respiration which does not coupled with ATP production (V_2_) but inhibits ADP-dependent respiration (V_3_). Moreover, PolyP can stimulate F_0_F_1_-ATPase activity in the presence of ATP and, importantly, can be hydrolyzed in this enzyme instead of ATP. Furthermore, PolyP can be produced in mitochondria in the presence of substrates for respiration and phosphate by the F_0_F_1_-ATPsynthase. Thus, polyP is an energy molecule in mammalian cells which can be produced and hydrolyzed in the mitochondrial F_0_F_1_-ATPsynthase.

## Introduction

Inorganic polyphosphate (polyP) is the oldest polymer found in living nature. It consists of a large number of orthophosphate residues which are connected by high energy bonds. In lower organisms, it is used for many functions and kept at very high concentrations as an energy source [1,2]. Despite the efforts made in the last 30 years, analogs of the two major enzymes used in bacteria or yeast for production or consumption of polyP — polyphosphate kinase and polyphosphatase, respectively, have not yet been identified in higher organisms.

Regardless of the relatively low concentration of polyP in mammalian cells (up to 100 µM) compared with those in yeast or bacteria, this polymer has been shown to play an important role in physiology as well as in the development of different pathologies in higher organisms [3–5]. Thus, polyP plays an important role in the mechanism of regulation of cell death [4,6] and in the development of many pathological processes, e.g. abnormal levels of polyP have been found in cancer cells [7,8] and in neurons with familial forms (PINK1, LRRK2) of Parkinson's disease [9]. Interestingly, a transgenic mice overexpressing exopolyphosphatase from yeast, which ultimately reduce polyP to very low or almost non-detectable levels of this polymer in all cells, exhibited a phenotype with enhanced lactic acid production and reduced ATP levels [10].

As a polymer, polyP acts as a scaffold for aggregation of misfolded proteins in neurodegeneration [11,12]. Moreover, the similarity in chemical structure between ATP (three orthophosphates) and polyP (multiple orthophosphates) spreads over to further similarity in the function they both exert in physiology. Thus, polyP plays an important signaling role in the mammalian brain: located to and released from ATP-containing vesicles [13]; it activates neurons and astrocytes through P2Y1 (known to be mostly specific to ATP and ADP) purinoreceptors [14,15]. Interestingly, polyP is shown to be an energy source for extracellular ATP production [16,17]. Previously, we have shown that polyP is playing an important role in mitochondrial function [18–20]. On one hand, a removal of polyP from mitochondria by the expression of mitochondrially targeted yeast polyphosphatase protected cells against calcium-induced cell death, but on the other led to the disruption of the mitochondrial energy metabolism [18]. Production of polyP in mitochondria is dependent on their energy state and, interestingly, is sensitive to the same substrates and inhibitors as the process of production of ATP [19]. An importance of polyP for the proper function of the mitochondrial NAD kinase [21,22] has been shown, which potentially affects the total mitochondrial metabolism. The role of polyP as an energy source for the ATPase has been already discussed [23]. However, there are still a large number of open questions concerning the role of polyP in energy metabolism [23]: (a) it is still not clear whether polyP could act as an energy molecule in mammalian cells, being utilized in the various types of ATPases; (b) how is polyP produced or elongated in mitochondria and how this polymer affects mitochondrial respiration and ultimately oxidative phosphorylation.

In the present study, we have investigated the effects of polyP on mitochondrial respiration and oxidative phosphorylation. In particular, we tested whether polyP could be produced or hydrolyzed in mitochondrial F_0_F_1_-ATPase. We have found that polyP, ATP alike, can be produced in the F_0_F_1_ATP synthase and as well can be used as an energy source in the F_0_F_1_-ATPase. Importantly, polyP interacts with ADP in the processes of mitochondrial respiration and oxidative phosphorylation. Overall, the abundance of polyP dramatically increases ATP production in mitochondria.

## Materials and methods

### Isolation of mitochondria

Mitochondria were isolated from the liver of Sprague–Dawley rats (150–200 g, UCL breeding colony) as described in [20] with some modifications. Experimental procedures were performed in compliance with the United Kingdom Animals (Scientific Procedures) Act of 1986. For experiments which produced in the UCL, animal studies were approved by the UCL ethical committee and performed under a U.K. Home Office project license. For isolation of mitochondria in the laboratory of Experimental Biophysics, Centre for Advanced Technologies, Tashkent, Uzbekistan, all animal work was approved by the Institutional ethical committee in compliance of the Republic Uzbekistan legislation. Briefly, the animals were euthanized by cervical dislocation, the liver was taken out, homogenized and re-suspended in isolation buffer (300 mM sucrose, 2 mM EDTA, and 5 mM Tris–HCl, pH 7.4). Mitochondria were isolated by differential centrifugation at +1°C temperature. Nuclei and intact cells were centrifuged for 12 min at 600×***g***. The resulting supernatant was centrifuged for 18 min at 6000×***g***. Mitochondria (resulting pellet) were re-suspended in 500 µl of isolation buffer without EDTA and put on ice. Protein content was measured by the Biuret test with BSA as the standard.

### Preparation of sub-mitochondrial particles

Sub-mitochondrial particles were produced by the method of freezing–thawing [24]. Freshly isolated mitochondria, were frozen in −20°C until the day of the experiment. The procedure of freezing–thawing creates a rupture in mitochondrial membranes, creating sub-mitochondrial particles. It is a very convenient model to study the ATP-hydrolyzing function of ATP-synthase as far as during membrane rupture and re-bounding membrane potential vanishing and concomitantly ATP-synthase cannot work as a synthase enzyme. On the contrary, the absence of membrane potential creates good conditions for ATP-hydrolyzing activity of the enzyme in case of substrate presence. After thawing procedure, the protein content was re-measured by the Biuret procedure with BSA as the standard.

### Measurement of ATPase activity

A method based on the fact that ATP hydrolysis at pH values close to neutral leads to the release of H^+^ ions in the sample due to differences in the pKa of dissociating groups of substrate (ATP) and reaction products (ADP and P_i_). Detection of hydrolysis was performed as described in [25], in the following medium: KСl 0.1 М, EDTA 50 μM, MgCl_2_ 2 mМ, Tris–HCl 5 mМ (рН 8.0) with the help of a pH meter at room temperature. Recording of the experiment starts after the addition of sub-mitochondrial particles to the media, which contained ATP, polyP or neither (control). A silicone stirrer was used to continuously mix the recording solution during the experiments. For the experiments, 100 mM Mg-ATP stock solution, pH 8, was used. Kinetics of pH shifts during the first 2 min of the experiment was used for calculations. At the end of each experiment, 200 nM of HCl was added for calibration.

### Oxygen consumption

Oxygen consumption was measured with the help of MitoCell S200 micro respirometry system based on Clark-type oxygen electrode (Strathkelvin Instruments, North Lankarshire, Scotland). The rate of oxygen consumption was measured over time, with the amount of added ADP — 200 µM, and CCCP 5 µM. The respiratory control parameter was calculated as the ratio of metabolic States V_3_ to V_4_ (V_3_/V_4_). **V_2_** or **V_sub_** — mitochondrial respiration rate in the presence of substrates without ADP; **V_3_** — mitochondrial respiration rate activated by the addition of ADP; **V_4_** — mitochondrial respiration rate in the presence of substrates after all added ADP were converted into ATP; **V_CCCP_** — maximal respiration rate in presence of uncoupler CCCP.

ADP/O was calculated as the ratio of 200 µM of ADP (converted by mitochondria to ATP) to the amount of oxygen (µg) used during the oxidative phosphorylation process (V_3_). Measurements were carried out in the incubation medium: 120 mM KCl, 5 mM glutamate, 5 mM malate, 10 mM Tris–HCl, 1 mM KH_2_PO_4_, 1 mM EGTA, pH 7.1. All measurements were carried out at room temperature (25°C), under continuous stirring. Mitochondria were added to the chamber at a concentration of 1 mg protein/ml.

### Measurement of DAPI-polyP fluorescence in mitochondria

Experiments were performed on Cary Eclipse Fluorescence Spectrophotometer (Agilent Technologies, U.S.A.), using the method described in [19,26], with some modifications. Mitochondria were loaded with 60 µM DAPI (4′, 6-diamidine-2-phenylindole), (Molecular Probes, Eugene, OR) and incubated for 40 min on ice. For the registration of the DAPI-polyP signal, the suspension of mitochondria, loaded with DAPI, was excited at 405 nm and the emission spectra were collected at 505 nm. Mitochondria were added to the sample at a concentration of 0.5 mg/ml. All experiments were carried out at 25°C, in 3 ml quartz cuvettes, with constant stirring in incubation media: 120 mM KCl, 5 mM glutamate, 5 mM malate, 10 mM Tris–HCl, 1 mM KH_2_PO_4_, 1 mM EGTA, pH 7.1.

### ATPase enzyme activity

ATP synthase enzyme activity was measured using an ATP synthase microplate kit (Abcam) according to the manufacturer's protocol. Briefly, brain mitochondria were isolated, lysed and the F_0_F_1_-ATPase was immunocaptured in the 96-well microplate supplied with the kit. The hydrolysis of ATP to ADP (or hydrolysis of polyP) is coupled to the oxidation of NADH to NAD^+^, which could be detected by a decrease in absorbance at 340 nm (30°C). The polyP was added immediately before measurements, absorbances at 340 nm were taken (F_0_F_1_-ATPase) in the presence/absence of different lengths/concentration of polyP, or in the presence/absence of oligomycin, and results were compared against control wells containing mitochondria only, in the same way as described before [27].

### Statistical analysis

Statistical analysis and exponential curve fitting were performed using Origin 8.6 software (Microcal Software Inc., Northampton, MA). Results were expressed as mean ± S.E.M. To determine the statistical significance of the results One-Way ANOVA and two-tailed *t*-test were performed.

## Results

### Polyp activates ADP-independent but inhibit ADP-dependent respiration

**Figure 1.**
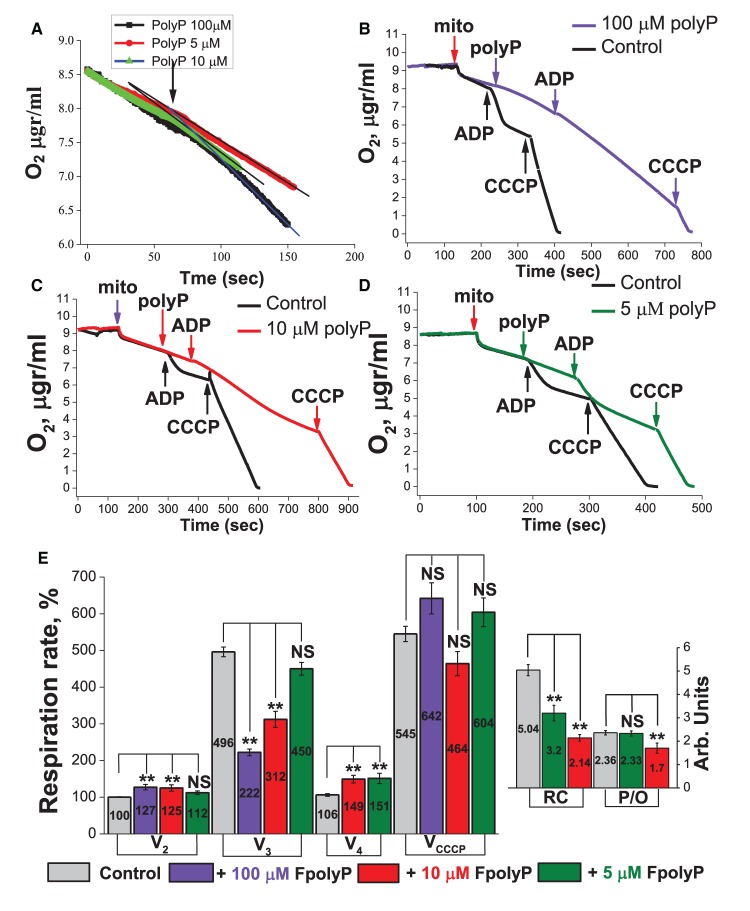
Effect of polyP on mitochondrial respiration. (**А**) Application of 100, 10 and 5 µM of polyP caused activation of mitochondrial respiration V_2_ (V_sub_); (**B**) application of 100 µM of polyP, decreased effect of ADP (V_3_), eliminated V_4_ state and did not change the effect of CCCP, compare to control; (**C**) application of 10 µM of polyP activated the V_2_ (V_sub_) state comparable to 100 µM, (**D**) application of 5 µM of polyP caused activation of mitochondrial respiration in V_2_ (V_sub_), respiration rate V_3_ state was decreased compared with control. (**E**) PolyP-induced changes in the respiration rate of isolated mitochondria. The respiration rates in V_2_ of control samples was taken as 100%. Application of polyP caused concentration-dependent decrease in **RC** and **P/O** ratios compared with control. Respiration rate in % and **RC** and **P/O** ratios in arbitrary units, were placed in the center of each bar chart. *n* = 110 for V_2_ in control, *n* = 55 for control (all other states of respiration), *n* = 11 for 100 µM of polyP, *n* = 17 for 10 µM of polyP, *n* = 12 for 5 µM of polyP. **P < 0.01, NS, not significant.

Previously, it was shown that the level of inorganic polyphosphates in the cells is directly coupled to the mitochondrial metabolism [19]. However, the role of polyP in mitochondrial respiration and oxidative phosphorylation remains unclear. For the mitochondrial respiration experiments, we used three different concentrations of polyP (fixed number of 100 orthophosphates) — 100, 10 and 5 µM. Importantly, application of all these concentrations activated mitochondrial substrate respiration (V_2_ state, Figure 1A,B,E) that possibly can be explained by membrane modifying properties of polyP [20] or by activation of polyP synthesis and activation of respiration in ADP-dependent way. In contrast, exposure of mitochondria to 100 µM polyP inhibited the ADP-dependent respiration (V_3_ state); thus, respiration rate in V_3_ state in the presence of 100 µM of polyP decreased twice compared with control (Figure 1A,B). Importantly, high concentration of polyP inhibited transition into V_4_ (then all added ADP converted into ATP in the process of oxidative phosphorylation), it was not observed even after 317 s of the experiment (we waited the moment of transition into V_4_ state ≈8 times longer compared with control — see Figure 1B). Application of mitochondrial uncoupler CCCP (5 µM) caused a substantial increase in the rate of mitochondrial respiration in the presence of polyP which was comparable to control (Figure 1A,B). As far as there was no V_4_ state in the presence of 100 µM of polyP, we were unable to calculate RC and P/O ratio for these experiments.

The effects of lower concentrations of polyP (10 and 5 µM) were smaller in different respiration states. Application of 10 and 5 µM polyP is also increased the rate of respiration in V_2_ state on 25.07% and 12.2%, respectively (Figure 1A,C,E for 10 µM polyP and Figure 1A,D,E for 5 µM). It led to dramatic decrease in the rate of ADP-dependent respiration (V_3_) in the presence of 10 µM but not 5 µM of polyP (Figure 1C,E for 10 µM polyP and Figure 1D,E for 5 µM). Importantly, ADP-independent respiration in V_4_ state was higher in experiments with 5 and 10 µM polyP (Figure 1C–E). As a result, polyP-induced concentration-dependent decrease in the Respiration Control ratio (V_3_/V_4_, Figure 1E) and in the ADP/O (P/O) ratio. Thus, polyP increase ADP-independent respiration but inhibit V_3_ that suggest the effect of polyP on the oxidative phosphorylation it is more likely because polyP can activate respiration in the absence of ADP due to activation of polyP synthesis which induce leak respiration in the same way as ADP. However, polyP compete with ADP on the F0-F1 ATPAse that lead to decrease in ADP/O ratio.

### Polyp can activate F_0_F_1_-ATPase proton pump with or without ATP

Previously, we have shown [19] that production of polyP depends on the activity of mitochondrial F_1_F_0_ ATP synthase. This enzyme can work in two directions — ATP synthesis utilizing the transmembrane proton gradient and function as a proton pump using ATP hydrolysis [28]. To find out whether polyP could be produced or hydrolyzed in F_1_F_0_ ATP synthase, we measured the activity of this enzyme. Most of the methods of assessment of F_1_F_0_ ATPase activity are based on the measurement of Pi release from ATP. Considering this these measurements could not be applied in our experiments due to the interference of polyP with Pi dependent reagents. To avoid it, we measured pH changes induced by the transport of H^+^ from sub-mitochondrial particles by F_1_F_0_ ATPase [29].

**Figure 2.**
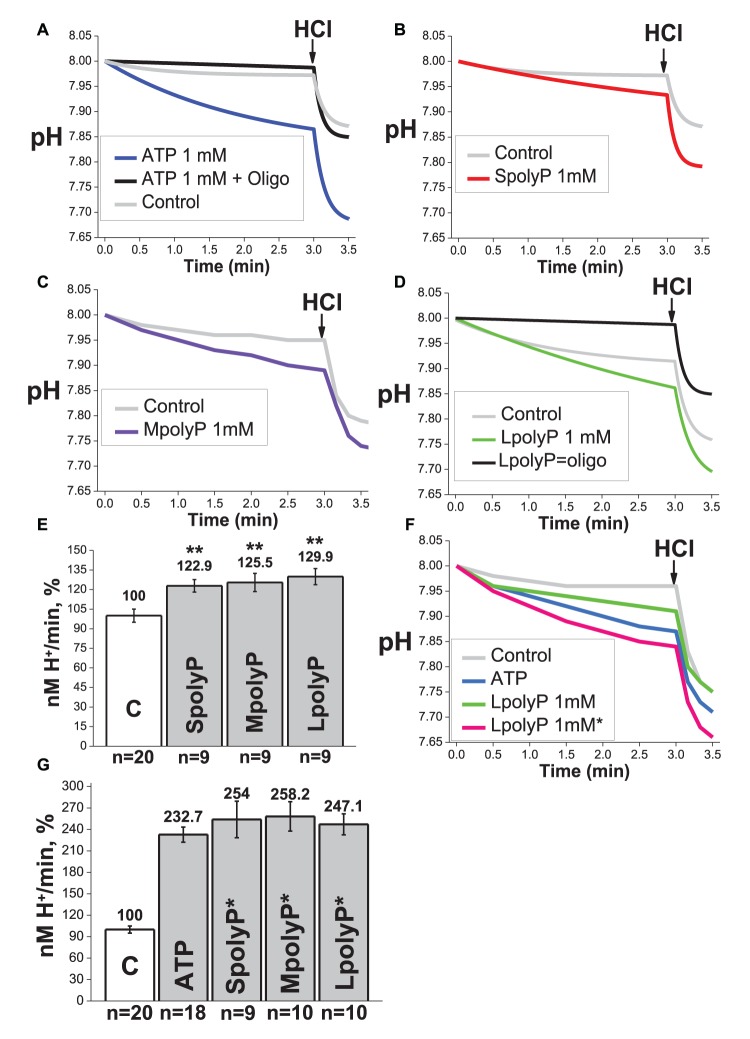
Hydrolyzing activity of mitochondrial F_1_F_0_ ATP-synthase in the presence of inorganic polyphosphates. (A) ATPase activity of F_1_F_0_ in the presence of 1 mM of ATP with or without 2 µg/ml oligomycin; (**B–E**) presence of 1 mM of S, M and LpolyP caused activation of hydrolyzing activity of F_1_F_0_ in the absence of ATP on 22.9 ± 4.8; 25.5 ± 7 and 29.9 ± 6.2%, respectively, and can be blocked by 2 µg/ml oligomycin (**D**); (**F**,**G**) co-application of ATP and polyP increased the rate of acidulation of the buffer additively to 254 ± 25.6%; 258.2 ± 20.5% and 247.1 ± 14.7 for S polyP, M polyP and L polyP, respectively. **P < 0.01.

We used three types of polyP — SpolyP, MpolyP and LpolyP, with 14, 60 and 130 ortophosphates length, respectively. The presence of 1 mM ATP increased the rate of acidulation of the buffer on 132.7 ± 10.6% (Figure 2A, *n *= 18). Importantly, these changes can be blocked by an inhibitor of F_1_F_0_ ATPase—2 µg/ml of oligomycin (Figure 2A, *n *= 5). Addition of polyP 14, 60 and 130 caused an increase in acidification rate on 22.9 ± 4.8; 25.5 ± 7 and 29.9 ± 6.2%, respectively (Figure 2A–D) and effect of polyPs on the activity of F_1_F_0_ ATPase also could be blocked by 2 µg/ml oligomycin (Figure 2D). These results indicate that in the absence of ATP, polyP act as a substrate for F_1_F_0_ ATPase. The rate of hydrolysis was dependent on the type of polyP and was higher with the elongation of the polymer (Figure 2B,D). Interestingly, the co-application of ATP and polyP increased the rate of acidulation of the buffer additively to 254 ± 25.6%; 258.2 ± 20.5% and 247.1 ± 14.7 for SpolyP, MpolyP and LpolyP, respectively (Figure 2A,B).

### Polyp can be hydrolyzed in F_0_F_1_-ATPase

**Figure 3.**
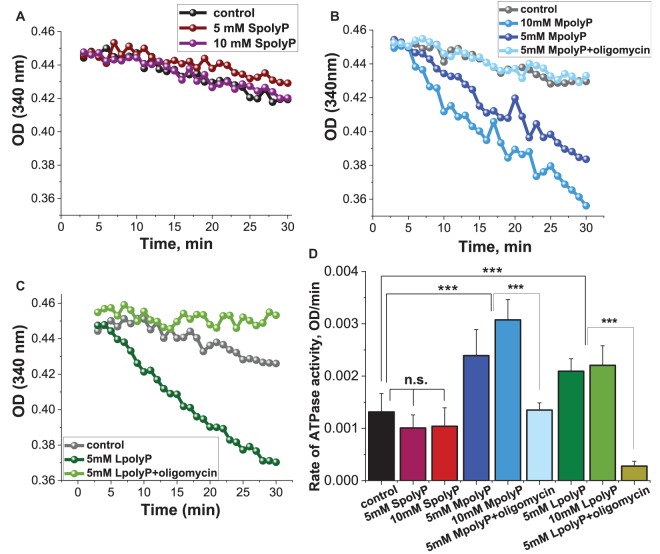
PolyP is a substitute for ATP in a F0F1-ATPase assay. (**A**) Short-chain polyP did not increase the ATPase activity, whereas MpolyP (**B**) and LpolyP (**C**) at both 5 and 10 mM concentrations, significantly decreased the absorbance at 340 nm overtime, corresponding to an increased consumption of polyP by the F0F1-ATPase (raw traces). 2 µg/ml oligomycin inhibit the effects of MpolyP (**B**) or LpolyP (**C**) on the F_0_F_1_-ATPase activity. (**D**) Quantification diagram of the rate of ATPase activity, shown as OD/min of the experiments, partially represented on (**A C**). ***, *P *< 0.0001.

To verify whether polyP regulates ATP synthase activity, rat brain mitochondria were isolated and the F_0_F_1_-ATPase immunocaptured in the wells of a microplate as described in the materials and methods section. This assay allows measurements of polyP effects on the ATPase activity in the absence of any other mitochondrial protein. Different lengths of polyP were then added to the wells immediately before absorbance was measured at 340 nm. The effects of polyP on the activity of F_0_F_1_-ATPase were dependent on the length of the polymer. Thus, the addition of 5 or 10 mM SpolyP instead of ATP had no significant effect of the enzyme activity (Figure 3A,D), however, MpolyP or LpolyP both led to a significant increase in F_0_F_1_-ATPase activity in concentrations of 5 and 10 mM when compared with control wells (100 ± 8%; Figure 3A; *n *= 4 experiments). Importantly, the polyP-induced activity of F_0_F_1_-ATPase could be blocked by 2 µg/ml oligomycin (Figure 3B,C,D). This assay provided evidence that polyP could be directly hydrolyzed by the F_0_F_1_-ATPase.

### Polyp can be produced in F_0_F_1_-ATP synthase of mitochondria

**Figure 4.**
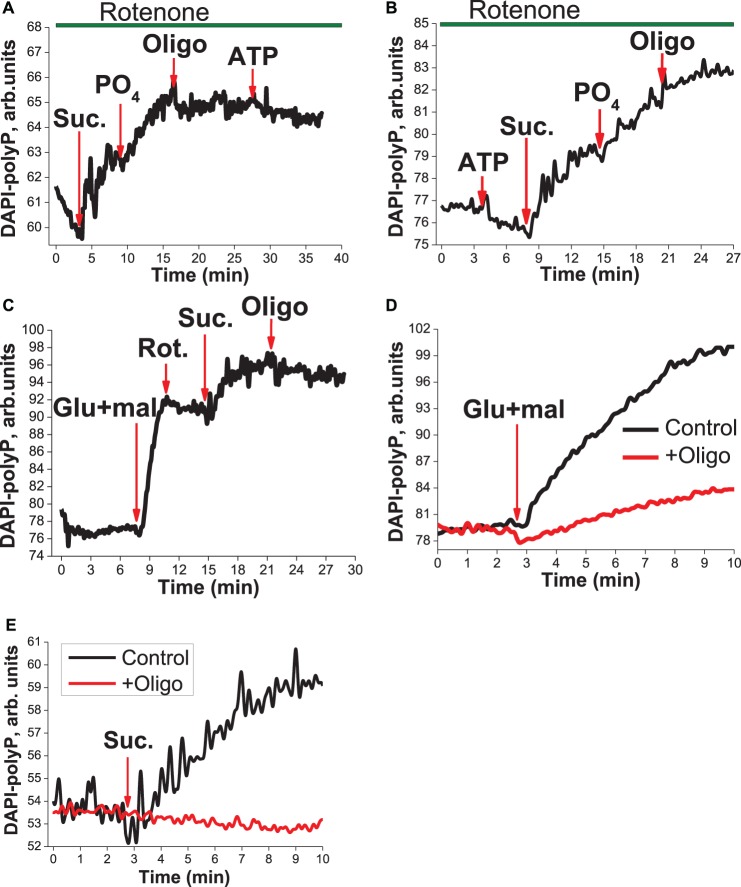
Production of polyP in isolated mitochondria. Measurements of polyP in isolated mitochondria using polyP-DAPI fluorescence. (**A**) Application of 5 mM succinate in the presence of rotenone (10 µM) induce increase in polyP level, which can be promoted by additional PO_4_ and blocked by oligomycin (2 µg/ml). Importantly, the level of polyP in mitochondria was independent of the application of ATP before (**B**) or after oligomycin (**A**). (**C**) The polyP level in mitochondria can be increased by application of substrates of complex I 5 mM glutamate/5 mM malate, inhibited by rotenone (10 µM) and further activated by 5 mM succinate. (**D**–**E**) Effects of substrates (D-5 mM glutamate/5 mM malate; E-5 mM succinate) on polyP cold be blocked by oligomycin (2 µg/ml).

To investigate the metabolism of polyP we used isolated mitochondria loaded with fluorescent indicator DAPI. Mitochondria in the presence of an inhibitor of mitochondrial complex I — 5 µM rotenone (no respiration) — showed no production of polyP (Figure 4A). Application of substrate for complex II—5 mM succinate— restored respiration and transmembrane potential in mitochondria and in our experiments it dramatically increased the level of polyP in isolated mitochondria (Figure 4A). Additional PO_4_ (1 mM) induced further rise in DAPI fluorescence, which could be blocked by the application of inhibitor of F_0_F_1_-ATPase oligomycin (2 µg/ml) (Figure 4A, *n *= 7). However, based on the inhibition of polyP production with oligomycin we cannot exclude that initially F_0_F_1_-ATPase produced ATP, and, this ATP was used for the production of polyP. To test it, we added ATP in the end of the experiment and we have not observed any rise in DAPI fluorescence, confirming that ATP does not participate in polyP production. Application of ATP in the beginning of the experiment before oligomycin also did not induce any rise in polyP level (Figure 4B, *n *= 8) suggesting that hydrolysis of ATP in ATPase has no effect on the polyP production in mitochondria. The level of polyP in isolated mitochondria could be increased by activation of mitochondrial respiration by application of substrates for complex I (5 mM glutamate/5 mM malate), inhibited by 5 µM rotenone and reactivated by 5 mM succinate (Figure 4C; *n *= 4). All these effects also were dependent on the presence of 2 µM oligomycin (Figure 4C–E; *n *= 4). It should be noted that the preincubation of mitochondria with oligomycin (3–5 min) completely prevented glutamate/malate- (Figure 4D) or succinate-induced polyP production (Figure 4E).

## Discussion

Here, we found that polyP can be used as an energy source for the F_0_F_1_-ATPase to pump protons. This was proven in our experiments by two independent methods. Importantly, polyP can be used by the ATPase alone, or as well in combination with ATP when effects of ATP and polyP do not inhibit each other. Our data are in agreement with previously published data on the effect of polyP on the activity of plasma membrane Ca^2+^ATPase [30]. Although the authors suggest different mechanism. In their study, the activation of plasma membrane Ca^2+^ATPase can be explained by the ability of this calcium pump to work as polyphosphate kinase. In contrast, we demonstrated before that polyP cannot activate calcium translocation in sarcoplasmic–endoplasmic reticulum Ca^2+^-ATPase (SERCA) [19]. However, we can suggest that Mg^2+^ dependence of these enzymes (F_0_F_1_-ATPase and plasma membrane Ca^2+^ pump are magnesium independent, but not SERCA) can allow for magnesium-dependent enzymes could use only ATP (which in the cell is predominately found in the form of Mg salt) while others could also use polyP.

Importantly, polyP could not only be utilized in F_0_F_1_-ATPase, but also be produced in this enzyme in the same way as ATP. Previously, we have demonstrated that in intact cells and in isolated mitochondria, the production of polyP is dependent on mitochondrial substrates and could be blocked by inhibitor of F_0_F_1_-ATPsynthase oligomycin [19]. However, these data could not exclude that polyP can be produced dependent on ATP, which is synthetized in F_0_F_1_-ATPsynthase. Our data strongly implicate that in isolated mitochondria application of ATP before or after oligomycin did not change polyP concentration that can exclude ATP as an intermediate in polyP production and prove that it produced in F_0_F_1_-ATPase.

It also should be noticed that we observed the activity of F_0_F_1_-ATPase in millimolar concentration (Figure 2 and 3). Although the average polyP concentration in the brain is 50–70 µM [3], polyP is highly compartmentalized [13,15,18] and concentration of polyP in some of the compartments (such as mitochondria) can reach millimolar level.

The effects of polyP in micromolar range in experiments with mitochondrial respiration indicate that it can be used for further polyP synthesis of polymer elongation.

One of the open questions left is how polyP and ATP interact/compete in the ATPase. According to the ATPase activity measurements, ATP is preferably the more effective substrate for the ATPase (Figure 2 and 3). However, reduction in the efficiency of oxidative phosphorylation in our respiratory experiments (ADP/O coefficient) in the presence of high concentrations of polyP suggests that a competing process might take place between the production of polyP and the consumption of ADP in this enzyme for the production of ATP.

Despite the higher activity of the ATPases with ATP, the ability of polyP to be hydrolyzed by the F_0_F_1_-ATPase and the plasma membrane Ca^2+^ATPase can be used as an energy source in ischemic conditions or in other conditions of substantial energy deprivation [31,32].

## Open Access

Open access for this article was enabled by the participation of University College London in an all-inclusive *Read & Publish* pilot with Portland Press and the Biochemical Society under a transformative agreement with JISC.

## Abbreviations

DAPI-4′: 6-diamidine-2-phenylindole
FpolyP: 100 orthophosphate residues
PolyP: inorganic polyphosphate
SERCA: sarcoplasmic–endoplasmic reticulum Ca^2+^-ATPase
